# Ethical challenges faced by French military doctors deployed in the Sahel (Operation Barkhane): a qualitative study

**DOI:** 10.1186/s12910-021-00723-2

**Published:** 2021-11-19

**Authors:** Antoine Lamblin, Clément Derkenne, Marion Trousselard, Marie-Ange Einaudi

**Affiliations:** 1 Anaesthesiology Department, Desgenettes Military Teaching Hospital, 108 boulevard Pinel, 69003 Lyon, France; 2 Anaesthesiology and Intensive Care Department, Edouard Herriot University Hospital, Hospices Civils de Lyon, 5 Place d’Arsonval, 69003 Lyon, France; 3 UMR 7268 ADéS Aix-Marseille Université, EFS, CNRS, Espace Ethique Méditerranéen, Efaculté de Médecine de Marseille, Timone University Hospital, 27 Boulevard Jean Moulin, 13005 Marseille, France; 4 Emergency Medical Department, Paris Fire Brigade, 1, Place Jules Renard, 75017 Paris, France; 5 Neurosciences and Cognitive Sciences, French Armed Forces Biomedical Research Institute, D19, 91220 Brétigny-sur-Orge, France; 6 French Military Health Service Academy, Ecole du Val de Grâce, 1 Place Alphonse Laveran, 75005 Paris, France; 7 Lorraine University, APEMAC/EPSAM - EA 4360, Metz, France

**Keywords:** Military medical ethics, Ethics, French military health service, War ethics, Law of armed conflict, Qualitative research

## Abstract

**Background:**

French military doctors are currently deployed in the Sahel to support the armed forces of Operation Barkhane, in medical or surgical units. As well as supporting French soldiers, their other missions are diverse and complex: medical assistance to civilians and persons under control (PUC), advice to commanding officers. These tasks can create ethical dilemmas when decisions are forced upon doctors that may be in conflict with medical values or fundamental principles. Little is known about the specific dilemmas experienced by French military doctors in overseas operations. We therefore conducted a qualitative study among doctors and surgeons recently deployed to the Sahel to explore and better understand this question.

**Method:**

Semi-structured, face-to-face interviews were conducted with 20 French military doctors or surgeons deployed since January 2016 in medical or surgical facilities in Mali and Chad.

**Results:**

All interviewed doctors reported having faced several ethical dilemmas during missions. All reported dilemmas involved the treatment of civilians (while delivering community medical assistance) or of PUC. The dilemmas involved choices as to which patients to treat, the use of care as a means to an end by military authorities, and the level of care attainable in the absence of any possible hospital follow-up. Questions of delivering care at the risk of their own safety or the mission’s and of treating openly hostile patients were also brought up. Several dilemmas stemmed from the dual loyalty problem, namely the conflict between military doctors’ duty of care to patients and to the military institution, but this was not the only factor involved. Contextual factors (restricted resources and security constraints) and psychological factors (especially hostility towards the enemy) were also associated with many of the reported dilemmas.

**Conclusion:**

This is the first reported study focusing on the ethical dilemmas encountered by French military doctors in overseas operations. It provides unique insights into their ethical experiences and should prove useful in improving operational training for healthcare personnel deployed on overseas missions.

## Background

The French army has been engaged in Operation Barkhane since 1 August 2014, the objective being to help partner states in the G5 Sahel (Mali, Niger, Chad, Mauritania, Burkina-Faso) develop the security capabilities to deal independently with the threat of Islamist terrorism in a comprehensive approach also involving political and economic development. More than 5000 military personnel are deployed in Operation Barkhane. Two hundred men and women from the French Military Health Service (*Service de Santé des Armées*, SSA) provide medical support, organized as recommended in NATO’s Allied Joint Doctrine for Medical Support [1]. Thirty *Role 1* medical teams (consisting of a general practitioner and paramedics) are present in the theater, along with three *Role 2* surgical teams (damage control surgery to stabilize patients) [2].

Military doctors have many duties in overseas operations, the foremost being medical support for Barkhane forces and treating injured French personnel [3]. Other missions include contributing to the direction and planning of operations by providing optimal medical support, preventive medicine, medical support for G5 Sahel and MINUSMA (*Mission multidimensionnelle intégrée des Nations unies pour la stabilisation au Mali*, United Nations Multidimensional Integrated Stabilization Mission in Mali) armed forces, medical training for G5 Sahel forces, and providing medical assistance to civilians (MAC).

MAC represents a considerable part of medical teams’ activities and accounts for more than 85% of SSA expenses [4]. In 2020, French medical teams performed 15,199 medical and surgical consultations, 104,795 paramedical procedures (e.g. dressings) and 718 surgical interventions for civilian patients in the Sahel. It is delivered free of charge and is a component of civil-military cooperation, to make the military’s presence more acceptable to civilians by establishing links with local actors, thereby contributing to rebuilding the country and restoring peace [5]. Chad and Mali, where most French military operations are conducted, have two of the weakest health systems in the world. Human and material healthcare resources are scarce, especially in terms of hospital capacity. In Mali in 2010 for example, there was one hospital bed per 10,000 inhabitants (compared with a worldwide average that year of 26 beds per 10,000 inhabitants), and these beds are mainly located in the capital, far from the areas where the French army operates [6]. Because of safety concerns, very few NGOs other than the International Red Cross operate in Mali. The French army interacts occasionally with the dispensaries run by the Red Cross in several provinces to recruit patients for MAC. There is therefore a gulf in the standards of care provided by the SSA and the general health system in Mali, which often means that patients cannot be transferred to local structures. As a result, MAC can only include outpatient surgeries (e.g. inguinal hernia operations, appendectomies, hemithyroidectomies, etc.) and the treatment of simple medical pathologies (e.g. infectious diseases) to avoid saturating *Role 1* and *Role 2* medical and surgical facilities. Emergency surgery is also provided for civilians when possible, especially for collateral casualties defined as having suffered “unavoidable injuries (…) inflicted by belligerents during necessary military operations” [7].

Massive casualty incidents are not uncommon but are difficult to manage because of their suddenness, the number of casualties, and the severity and mutilating nature of the injuries. Patients have to be classified and triaged for evacuation to the surgical unit, where they are then triaged again for surgery [8]. Medical teams can also be called upon to evaluate and treat persons under control (PUC), detained for the safety of Barkhane forces or for the local population’s in the ongoing armed conflict. The challenge is to provide the same level of care as for other patients, ignoring the acts they are suspected of having committed, in keeping with international laws and medical ethics [9–11].

Practices have to be adapted to the security constraints and restrictions on human and material resources. Financial resources are also limited and restocking medical supplies and equipment is difficult to impossible, particularly in the most remote areas.

The conditions under which military doctors operate are unique. They report up two chains of command, one hierarchical, the other technical. As officers, they are hierarchically subordinate to the military command of their unit. The head of the technical hierarchy is the director of medical affairs (DMED), an experienced doctor based in command headquarters in N’Djamena (Chad), who advises the general in command of Operation Barkhane. The DMED also acts as a bridge between military authorities and medical teams along with the patient evacuation coordination cell (PECC) doctor [12].

This dual subordination and their multiple duties are fertile grounds for ethical dilemmas: patient management decisions are often ambiguous and involve possibly conflicting sets of values (and/or responsibilities and/or duties and/or commitments) [13]. Several studies conducted in other armed forces have investigated the ethical dilemmas faced by medical personnel on overseas operations or humanitarian military operations [14–20]. Other than the widely debated involvement of US medical personnel in interrogations [21, 22], the most commonly discussed ethical dilemmas involve the rationing of resources and inequalities in standards of care between fellow personnel and other categories of patients (civilians, prisoners, etc.). Results in the humanitarian medicine literature are similar [23, 24].

No study has ever been published on the ethical dilemmas faced by French military doctors in overseas operations. We studied their “ethical experience” during Operation Barkhane, which is the main overseas theater for French armed forces but also one of the largest ongoing deployments of military doctors worldwide. The main objective of this study was to analyze the ethical dilemmas and challenges reported to have been faced by French military doctors in Operation Barkhane since 2016, and identify the circumstances and situations in which they arose. The secondary objectives were to compare these dilemmas to those reported in the (mainly Anglo-Saxon) literature, and suggest possible improvements in ethical preparation and operational training in the French army.

## Material and methods

This was a qualitative observational study, based on semi-structured interviews, inspired by Miles and Huberman’s method of qualitative data analysis: data condensation and display (reduction, coding), formulating hypotheses, and verifying conclusions [25].

The inclusion criteria were that participants had (1) to be an active military doctor, (2) to have been involved in Operation Barkhane in *Role 1* or *Role 2* units since 1 January 2016. The participants were doctors with operational specialties (surgeons or anaesthesiologist-intensivists) practicing in military teaching hospitals in metropolitan France, or general practitioners in the French army. Military doctors who met the inclusion criteria were identified beforehand and contacted directly by email to explain the aims and methods of the study. A consent form was also provided. There was no pressure from superiors to participate; voluntary informed consent was sought from all participants and anonymity was guaranteed.

The interviews were conducted between May 2019 and January 2020, as recommended for qualitative research [26, 27]. An interview guide organized by theme was prepared beforehand, based on the literature, with open questions that the principal investigator and the participant were free to deviate from. The interview guide was reviewed by experts in qualitative research (Marie-Ange Einaudi, Aix-Marseille University, Bruno Décoret, Lyon University). Their comments were incorporated into the final interview guide, which had several parts: demographic characteristics, medical and operational experience, description of ethical dilemmas faced in overseas operations, methods of resolving or treating the dilemma, specific preparation or needs expressed regarding their management in overseas operations. An exploratory interview was performed to evaluate the quality of the interview guide, which was then reworked based on the participant’s comments. All interviews were conducted face-to-face, in French, by the study’s principal investigator (AL), at participants’ place of work. The interviews were recorded after written consent was provided for participation and for the audio recording of the interview. Notes were taken during the interview to record the principal investigator’s feelings and impressions. The interviews were confidential and no identifying details were recorded. Participants were assigned an inclusion number to ensure anonymity. Their ranks are not mentioned in the results.

All interviews were transcribed in full, then synthesized and coded using the Nvivo 10 software (QSR International Pty Ltd., Doncaster, Australia). Five interviews were coded independently by AL and CD, to establish a consensus set of codes and minimize the risk of investigator bias. This set was then used to code the remaining interviews and the codes were subsequently regrouped into subcategories.

The study was approved by the institutional review board of the French Society of Anesthesia and Intensive Care Medicine (*Société Française d'Anesthésie et de Réanimation*; IRB 00010254-2018-154). It was also approved by the research office of the French Military Health Service (*Direction Centrale du Service de Santé des Armées*) and registered with the French data protection authority (*Commission Nationale Informatique et Líbertés*).

## Results

Ten interviews were conducted with *Role 1* general practitioners (GPs), deployed in combat units, and 10 other interviews were performed with *Role 2* specialist doctors (SPs): six anesthesiologist-intensivists, two gastrointestinal surgeons, and two orthopedic surgeons. Demographic characteristics and interview lengths are summarized in Table 1. All participants had at least been involved in Operation Barkhane. All but two (n = 18, 90%) had last been deployed in the western theater (Mali), where most military operations are concentrated, the eastern theater (Chad) being the support base, where the command center of Operation Barkhane is located.

**Table 1:** Interview lengths, demographic characteristics and levels of experience for general and specialist doctors

Interview length *mean [range]*		GP n = 10	SP n = 10	All n = 20
		55.7 [43–59] min	48.7 [40–55] min	51.7 [40–59] min
Sex	*F*	6	1	7 (35)
	*M*	4	9	13 (65)
Age *years*	< *30*	1	0	1 (5)
	*31–35*	6	6	12 (60)
	*36–40*	2	1	3 (15)
	*41–45*	1	3	4 (20)
Number of Barkhane missions	*1*	5	3	8 (40)
	*2*	3	2	5 (25)
	*3 or more*	2	5	7 (35)
Number of non-Barkhane missions	*0*	1	1	2 (10)
	*1*	2	2	4 (20)
	*2*	3	4	7 (35)
	*3 or more*	4	3	7 (35)
Medical experience *years*	*1–5*	3	2	5 (25)
	*6–10*	4	4	8 (40)
	*11 or more*	3	4	7 (35)
Year of last deployment	*2016*	3	0	3 (15)
	*2017*	3	0	3 (15)
	*2018*	0	4	4 (20)
	*2019*	4	6	10 (50)
Location of last deployment	Barkhane East*	0	2	2 (10)
	Barkhane West**	10	8	18 (90)

Data are presented as mean [range] or number (percentage). *GP* general practitioner, *SP* specialist doctor

* Eastern theater (Chad) ** Western theater (Mali)

Within the dataset, we chose to highlight the ethical dilemmas experienced by participants using representative quotes. To ensure anonymity, respondents are referred to by their inclusion number (GP1, GP2 etc. for general practitioners and SP1, SP2 etc. for specialist doctors). All participants felt in retrospect that they had faced several ethical dilemmas when deployed in Operation Barkhane.

### Professional identity

Participants were asked to describe how they perceived their dual status as a doctor and a soldier in overseas operations. None of the respondents considered themselves combatants, stating that while they did carry weapons, this was only to defend themselves and not to take any active part in combat operations:

“It is questionable how much sense there is to carry a weapon and a stethoscope in the same bag. I see myself as back-up, not as a combatant. My weapon is only there for self-defense.” MG6

All respondents felt comfortable in their roles as caregivers and soldiers, even though they reported their position as being apart in operations, their primary mission being to provide medical support to Barkhane forces:

“We are appreciated for our true worth by the soldiers we work with. We are above all doctors, but in a setting that forces us to remember that we are also soldiers, at the service of power and politics”. SPE3

### Ethical dilemmas faced by participants in overseas operations

Thirty-six codes were identified and grouped into five topics that highlight the dilemmas encountered by respondents.

For several of the identified themes, we added in parentheses the factors and bioethical principles that we considered to be at odds with the dilemmas raised by the participants. Beneficence, non-maleficence and distributive justice are defined as described by Childress and Beauchamp [28]. Quality of life is defined according to the four-quadrant approach, i.e. it refers to the assessment of the type of life envisaged by the patient with the proposed treatment [29].

### Faced with the impossibility of treating all presenting patients, which ones should be chosen? (Resource rationing and distributive justice)

In providing MAC, five respondents (MG2,3,9 and SP7,9) reported that they had had to make difficult decisions regarding which patients to take care of. Choices were influenced by third parties, local civilian recruitment officers (LCROs) or nurses, who wanted patients to be sorted by perceived social value, sex or age criteria unfavourable to women and children and discriminatory.

“I had to deal with limited resources. On the first day, I have this image in my mind of me and my 25 consultation coupons in my hand and several hundred people around, and I had to choose. The Chadian nurse was saying: “you have to take local soldiers”, and I had 30 dying children.” MG2

Other respondents reported financial discrimination. Consultation coupons were bought by patients from LCROs, and only those with the means to pay could access MAC, which is supposed to be free.

Still in the context of delivering MAC, four specialist doctors (SP2–4,6) mentioned having had to abandon too severely ill patients despite having been able to treat them, because this would have consumed considerable human and material resources at the expense of the many more patients with more easily treated conditions.

“I once had a patient with an ulcerated hip eschar with bone exposure; we decided to not treat him even though he was young, because that would have led us into a treatment course that we would not have been able to complete, with significant personnel time and material costs”. SP6

Regarding the treatment of multiple battle casualties, several respondents reported having had to prioritize French patients or foreign armed forces personnel, based on utilitarian principles. These situations were not perceived as true dilemmas, as respondents considered their training in this area sufficient:

“Regarding mass casualties, dilemmas can easily arise, but in this case training is adequate, in that the collective interest should come before any individual interest to avoid dilemmas. This happened once during a sudden influx of French casualties. One was considered hopeless. We operated on him last. He was clearly in a critical condition. We first considered his case hopeless but then took care of him after reclassifying him as operable. This raised questions, particularly since this soldier was SSA personnel and several members of the medical team, myself in particular, knew him well.” SP1

The question of prioritization based on nationality during mass casualty events was raised by several participants (GP6, SP3,7). Although none were forced to choose between a French and foreign casualty (PUC or other), this possibility was mentioned as a potential dilemma between the principles of non-discrimination on one hand and of duty to fellow soldiers on the other. All respondents who raised this question believed that they would choose to treat the French casualty first, at equal severity or even if the French patient’s condition was less severe:

“The question came up of what choice we would make if two casualties arrived, between a French and an enemy patient. If their conditions were similar or even if the French patient’s condition was less severe, we would have operated on the French casualty first. Even if on normative or ethical grounds we’re told we shouldn’t, we would have done it anyway. Compromising a comrade’s functional outcome to treat an enemy casualty, that would not have gone down well with other soldiers on the scene and would have been difficult on a personal level.” SP7

One participant mentioned that triage for *Role 2* evacuations was performed upstream by the combatants themselves, who in practice prioritized French casualties over PUC or the members of foreign armed forces:

“In tactical MEDEVAC priorities from the field to the Role 2 unit, Barkhane soldiers come first. It goes without saying. There’s a form of informal discrimination.” SP3

### Should treatments be given if they are of no benefit to the patient, only to serve institutional military interests?

Almost all respondents mentioned being confronted with this dilemma during MAC missions (GP1–8, SP1,2,5,7,8). Most of these situations occurred in Barkhane’s western theater, where most so-called “opportunity” MAC missions are carried out (operations following reconnaissance missions for example, of limited duration), in contrast with the eastern theater where MAC missions have been in place already for several years, where continuity of care is guaranteed. These MAC interventions were described as being directed by military authorities, solely in their interests, to obtain information, facilitate diplomatic exchanges with village leaders or persons deemed “of interest” by the military, or as an opportunity to communicate with civilians and make the presence of French military forces more acceptable, in Mali in particular:

“Military authorities asked us for targeted medical operations to foster good relations and discussions. (…) So then this raises the question: why examine so-and-so who doesn’t really need it and not someone else in the village? GP1

Doctors in *Role 2* units described having been ordered by military or medical authorities to treat patients whose condition was beyond their treatment capacities, for diplomatic reasons or because they were relatives of local leaders. One participant (SPE2) described having been assigned to treat a civilian patient with severe burns (> 50%), consuming large amounts of medical supplies and leading to the cancellation of scheduled MAC surgeries for several days. The patient was transferred after several days to a local facility and died after a few hours because of inadequate care.

Some military doctors considered themselves commodified by military authorities, despite being well aware that the secondary benefits for the military of MAC missions is clearly part of the SSA’s doctrine [30]. Some went as far as describing opportunity MAC activities as worthless and questioned their soundness and utility. The lack of follow-up for chronic pathologies made them impossible to treat, and seemed contrary to the proper practice and very foundations of general medicine. In these circumstances, MAC was therefore described as being devoid of any medical value, particularly when it simply involved distributing drugs:

“The instructions we had from military authorities were to focus on quantity, see as many patients as possible. They had been on my case, they told me that I wasn’t going fast enough, that I should be seeing 70 patients in two hours. I disagreed. There should have been fewer people so as not to cut corners. Patients are well aware that if you just give them a box of pills, that’s not enough. For me, this may be naïve of me, but I was there for the patients. I know that MAC is politics to make the troops’ presence acceptable. No need for doctors in that.” GP7

The risk of interfering in local health systems or with non-governmental organizations was also raised as a potential hazard:

“In Mali, there are opportunity MAC operations where you go to hand out pills, you always wonder about medical legitimacy, especially in the desert. You tell yourself you’re going into a medical center bypassing what is going on at a local level, for very little benefit. Reasons for consulting, there were no real needs. I thought I would see poverty. There was a program run by the Red Cross. (…) There was no follow-up. We had to go for quantity. Time was limited and we were told that we had to see everyone that had turned up. The risk is that patients are not considered as humans but only in terms of the benefit they represent for the force.” GP6

One participant even mentioned the use by Barkhane forces of MAC as a means of coercion on local communities:

“There had been strikes among local civilian recruitment officers. Central command told us to cut off Role 2 MAC as retribution. There was a crisis meeting, and we were told that the first thing to do was to stop MAC. We didn’t do this.” GP9

### How to deliver healthcare when the team’s safety or one’s own is in danger? (Beneficence and security or operational constraints)

Several respondents (GP2,7 and SP3,10) raised the question of the therapeutic relationship with PUC, made difficult by security constraints. Doctors wore a balaclava, and patients were handcuffed, preventing any sort of patient-doctor reciprocity:

“You treat patients wearing a balaclava, masked. There can’t be any empathy. There is no therapeutic relationship on equal footing. The only thing these prisoners saw were masked individuals. Exchanges are poor. There is no reciprocity. All the non-verbal is attenuated or annihilated.” SP3

Three combat unit doctors (GP3,4,5) mentioned having been ordered not to treat injured or sick patients because of strong security constraints, the surroundings not having been secured. One doctor also described having had to refuse to treat a civilian patient with severe malaria who had broken into the French military base:

“They found one day in a tent a man in a confused state, with a temperature, probably malaria. He had no animosity toward the forces. He was incoherent. I would have liked to keep him on the base to rehydrate him, put him on a drip and treat him. He wasn’t all that young. The instructions, the orders that were given were to not keep him (…). They told me that he had broken in, that we could not keep him (…). So I just gave him an oral treatment, in spite of his vomiting.” GP7

A *Role 2* surgeon (SP6) reported having had to deal with a unique situation. While performing a hernia operation as part of MAC, the alarm for an airborne missile attack sounded. The instructions in this situation are to proceed immediately to a secure shelter outside the *Role 2* base. The question he asked himself was: “*should I go to the shelter and risk leaving the patient alone on the operating table?”.* He and the nurse anaesthetist finally decided to stay with the patient rather than shelter.

During reconnaissance missions, two GPs (GP6,9) had to make the difficult decision to not or minimally treat civilian victims of rebel attacks, whose village had been targeted in retaliation by terrorists while they were defenceless. They described having discovered these by chance in the middle of the desert. Without any means of evacuating them, the question was raised whether they should evacuate the injured themselves and end the mission. In both situations, after discussions with commanders, the decision was made to leave the injured and find another solution to evacuate them, with no guarantee that this would be done.

Many interviewed doctors had to decide whether to preserve limited medical and evacuation capacity for possible French casualties (GP2,6,9,10, SP2,5,8). They limited treatment for PUC or civilians to preserve their compatriots’ safety. This highlights the difference in standards of care between French casualties and others, creating a certain form of discrimination:

“On my first mission in Mali, the first two casualties we treated were enemy fighters who had come under fire from Barkhane forces during the night. There were operational constraints with convoys, ongoing operations. The difficulty was to work out how to evacuate these men who needed to be hospitalized in the Role 2 in Gao. This was problematic between the deputy DMED, myself and the Role 2 head, and the convoy personnel. We had to make plans to work out whether to delay the convoy to facilitate these men’s evacuation, who were enemies, or to keep going with the mission as a priority and evacuate the casualties secondarily. I fully understood at the time that the mission cannot be delayed for these casualties, but I saw that the evacuation conditions were very basic, and we wouldn’t have done that had they been French. I decided not to give them a transfusion, even though they would have needed one. I decided not to do it to save resources for Barkhane forces. Should I have done more for them, could we have optimized oxygen transport, hemodynamics, would this have allowed the patient who was subsequently amputated to keep his leg?” SP5

### What standard of care is achievable in the absence of adequate critical and/or follow-up care? (Non-maleficence, quality of life, intervention context)

Situations of this type only arose for patients whose evacuation to another treatment center other than the French *Role 1* or *Role 2* was impossible. These patients were civilians or PUC whose pathologies were so severe that non-intervention or decisions to limit treatment were considered, whereas in France they could have received the necessary treatment (GP1,5,10 and SP1–9).

“Within the limits of MAC, things can’t be done beyond reason. We ended up seeing a bedridden patient about 75 years old, who had had a stroke, probably some time ago, with pressure ulcers that had become infected. There are many things we could have done in France. Part of me wanted to take care of him. In Mali, there was nothing we could do.” GP1

“In Mali and Chad, we saw children who had been brought by their families for conditions that we could diagnose, such as for example a 5-year-old child who probably had very advanced stage Hodgkin’s lymphoma, with no possibility of treatment in the country. The families did not have the means to pay for treatment either. This created a dilemma because we could diagnose the condition but not treat it.” SP1

“We decided to stop treatment in a PUC. This was not easy for everyone. He had had several limbs amputated, a colostomy, a sacral pressure ulcer. He was dependent on opioids, with no chance of recovery because there was no possibility of rehabilitation. This was discussed as a group. Role 1 personnel did not understand the decision to limit treatment.” SP7

Some respondents related how they had had to downgrade the surgical management of PUC, since there was no possibility of transfer or evacuation (SP1,7,8):

“The third issue is with respect to PUC. It’s troubling from an ethical point of view. We had to deal with the fact that no evacuations or follow-up were possible, the inadequacy of the means available to treat certain PUC. This led us sometimes to make treatment decisions that were imposed on us by the situation, but that were not those the patients would have made. For example, one PUC subsequently had an arm amputated because we did not have the means to renew his treatment for long enough, a skin graft for example.” SP1

The question of the limited competencies of surgical teams was also mentioned as a source of potential ethical dilemmas, in particular for the treatment of children or pathologies outside the scope of surgery or anaesthesiology. Should operations be performed that would clearly involve overreaching their abilities?

“In Chad we had set a rule of not taking children less than 2 years old or 12 kg. I remember a girl who had arrived with dental cellulitis. She couldn’t open her mouth so the dentist could not do anything and sent her to me for surgical treatment. I refused because she was less than 2 years old, I did not have any equipment for difficult intubations like a fiberscope and paediatric resources were limited. I felt like I was kicking the can down the road relative to French standards, but considering the means at my disposal, this seemed like the right decision.” SP5

### Is it possible to treat patients that are openly hostile? (Impartiality and beneficence)

Several military doctors mentioned this problem as a source of ethical tension. They had moments of doubt when they had to treat PUC or patients who clearly demonstrated their hostility to French soldiers (GP1,3,4,8 and SP2,6,7,10):

“We did a MAC in a village. We were asked to do this 10 days after one of our armored medical vehicles had been blow up by an improvised explosive device. For my team, it was difficult to go and help a community suspected of having committed this act.” GP4

A sense of perspective was required to ignore the acts they were supposed to have committed and preserve a certain level of objectivity and impartiality, to provide a good standard of care to these patients:

“We use our resources, energy to treat these people who are potentially involved in actions against Barkhane forces, all this while being as objective as possible”.SP2

This attitude was all the more difficult to maintain that some combat personnel reproached the medical teams for treating PUC according to the same standards as French casualties (GP3,7,9, SP1,5,6). In some cases, these criticisms came directly from the respondents’ own subordinates (SP3,7,10):

“Regarding the treatment of one PUC, I heard from my subordinates: ‘why are we treating terrorists: they asked for it!’ Some thought that we should not treat them. There was also racism. Not everyone is well-meaning. That would soon come back to me and I would make a point with the team to remind everyone of the rules.” SP10

### Participants’ approaches to facing and resolving dilemmas

In making decisions, several participants reported having found answers in laws and regulations, particularly in the law of armed conflict (LOAC) for the treatment of PUC (GP1,10, SP1,10). The importance of collegiality in the decisions, when time constraints allowed for this, was highlighted by several respondents (GP1,5,6,10, SP1,2,4–10). This collegiality was part of a group reflection process between field doctors with the same healthcare roles or in a multidisciplinary approach involving for example the psychiatrist based in Mali or a doctor from a different specialty who was also present at the time of the situations discussed. Medical command (DMED and PECC) was also a privileged interlocutor in reaching decisions for 11 of the interviewees (GP2,3,6–8,10, SP3,5,7–9), some of whom mentioned nevertheless that this depended on the DMED and PECC doctor’s personality and positioning with respect to military command. Seven respondents declared having experienced a lack of support from their medical hierarchy, described as retreating from its responsibilities and simply applying orders received from command headquarters to the detriment of practitioners’ decisional autonomy.

These situations were also discussed with military commandment or the combat personnel themselves, in reaching decisions for seven respondents (GP2,4,5,10, SP1,8,10), or during debriefings for two others (GP7,9).

Ethical problems were shared between healthcare branches with paramedic personnel, to reach a decision (GP1,2,4–8,10, SP1,2,5,6,8–10), but also discussed during formal or informal debriefing sessions (GP3,4,6, SP2,6,8).

## Discussion

This is the first published study of the ethical dilemmas encountered by French medical doctors on overseas missions, during operation Barkhane, which is currently the French military’s main overseas engagement. The study group is representative of the different operational specialties involved. Participants reported many ethical dilemmas, with all interviewees reporting several such situations. The emergence of ethical dilemmas seems nevertheless to be correlated with patient status. Care provided to French soldiers was not reported as having been a source of ethical dilemmas or tensions, whereas treating PUC or civilians was.

GPs and SPs were both confronted with ethical dilemmas. These two categories have seldom been considered separately in studies. Rochon has shown that ethical dilemmas during the conflict in Afghanistan were more frequent among general practitioners than among specialist doctors because the former were more likely to experience command pressure [18], which was not the case in our study.

According to the World Medical Association, wartime ethical standards should be similar to those in peacetime [31], considerations that should be reflected in the practice of French military physicians. However, the present study highlights differences with civilian practice, because of the necessarily adaptations in field medicine to the context of any intervention and the dual status of military doctors. The ethical dilemmas reported by participants stemmed from the problem of dual loyalty, conditions of practice (limited resources or security constraints), or from issues of positioning with respect to PUC or openly hostile patients (impartiality). In all cases, patients’ interests, and therefore the principles of medical ethics, were at odds with other factors the doctors had to consider in their decisions.

American and British military forces established medical rules of eligibility during the former conflicts in Irak and Afghanistan, to regulate the flow of local nationals within their facilities. These rules took “military necessity” into account and classified patients based on their status rather than on their injuries [7]. Thus, civilians and non-coalition soldiers could only receive treatment if hospital beds were available and not required for the treatment of coalition soldiers, or if they were direct collateral casualties of coalition operations. No such rules have been established for Operation Barkhane, France having chosen to strictly follow international agreements. French military doctors abide therefore by their own specific training and the ethical rules of the LOAC, whereby patients are treated equally regardless of their status, based only on their medical needs [11, 32].

Dual loyalty is a concept that is often brought up in the literature [33, 34]. It stems from the idea that military doctors, who belong both to a medical organization and the military, are governed by two separate systems of values and obligations, and must consider the common good in their decisions, namely military interests and national security [35, 36]. Military doctors are therefore an integral part of an organization whose ultimate objective can be to undermine the heath or life of individuals to achieve its ends. This dual allegiance is not a unique feature of military doctors’ work; other types of doctors such as those working in the penal system are also concerned [37]. In fact, all medical practitioners have to deal with this tension between patient ethics and their duty to the community (for example not to squander expensive medical resources). But what is unique about military doctors’ work is that these two sometimes antagonistic interests can lead doctors who would abandon their duty of care to behave unethically, in violation of fundamental rights and human dignity, for instance by participating in acts of torture or interrogations, as reported on many occasions in the US literature [21, 22]. No acts of this type were reported by participants, but most experienced healthcare being used as a means to an end by the military establishment, when treating patients to obtain strategic information or for diplomatic objectives for example. This was perceived by several participants as a source of ethical tension in that they felt exploited and had to abandon their duty to the patient solely for the military’s benefit. Even if the official military doctrine clearly defines the role of MAC in increasing local acceptance of the armed forces, this must remain a secondary and not the primary objective [30].

Several authors mention the pressure experienced from commanding officers for reasons of military necessity, notably concerning the aptitude or not of sick or injured personnel to return to their posts [35, 38, 39]. In some instances, commanders would become impatient and pressurize doctors to speed up diagnosis or treatment, sometimes compromising the soldier's recovery and autonomy, and the practitioner's own decision-making autonomy [18]. Problems such as these were not reported by participants. Pressure both from military authorities and the medical hierarchy stemmed rather from requests to treat patients deemed of value to the military. What is most apparent in the interviews is the divergence of views between doctors and combat personnel on the quality and relevance of care for enemies. This can create a dissonance in military doctors leading to moral stress or unethical behaviour should doctors have to abandon deeply held ethical principles or commitments [40–42].

Military doctors are not neutral in performing their duties in the Sahel. Neutrality, one of the fundamental principles of humanitarian medical practice, would imply that medical assistance were delivered equally to all sides in the armed conflict. This is quite different from impartiality, wherein medical aid “must be provided solely on the basis of need, without discrimination” [43]. Military doctors operate in an institution whose values they have chosen to adhere to and bond with their fellow soldiers, developing a form of solidarity. All interviewed military doctors stated that they subscribed to the principles of the mission and felt comfortable in their roles as doctors and soldiers. Proximity with combat personnel in advanced posts and in the field promotes bonding and camaraderie. Military doctors cannot therefore be truly neutral, leading some to say that were they forced to choose between French and foreign casualties, they would favour the former. In caring for PUC, respondents acted with impartiality on an individual level, on the basis that human suffering should be treated equivalently in all patients, regardless of their status. The medical chain of command is supposed to guarantee independence and decisional autonomy. The military doctors in our study reported pressure being exerted by the medical hierarchy itself, at the behest of military authorities, a process some participants did not take well.

Many of the dilemmas described by participants concerned the context of interventions: limited resources, dealing with cultural differences (particularly in the sorting of patients for MAC), security constraints. These dilemmas are similar to those described in humanitarian military operations and humanitarian missions run by NGOs [24, 44, 45]. Military doctors have to make do with restricted human and material resources. This implies having to apply the principles of distributive justice for the greater good from a utilitarian perspective that may conflict with the principles of medical ethics. Barkhane military doctors must also consider the lack of medical facilities in the Sahel and the impossibility of any follow-up care. Decisions to limit treatment because follow-up was impossible were widely brought up by respondents as creating ethical dilemmas. These situations produce status-based care disparities, French military personnel being provided optimal care, since they can rapidly be evacuated to a healthcare facility in France.

The collective nature of decision making was a crucial feature reported by several participants as having helped them to manage or resolve the ethical dilemmas they faced. While the urgency of these decisions sometimes precludes group discussion, this should be encouraged as a way to share the burden of the decisions and limit the risk of moral stress even if the ultimate responsibility lies with the referring physician [46]. The PECC doctor and DMED, reachable at all times by phone, are privileged interlocutors provided the discussions are conducted on a collegial rather than a hierarchical basis. Collective discussions allow practitioners to share their doubts and anxieties and take a step back from often emotionally charged experiences they have encountered. Ethical discussions with other team members (nurses, medics, other doctors) or medical diplomacy also have a place once decisions have been made as a basis for further reflection and to suggest improvements for the future [47]. Post-deployment ethical debriefings should also be considered to bring teams that have faced ethical dilemmas on missions back together in the first few days after their return, with a counsellor trained in leading structured reflections and analyses of these dilemmas.

These results support the fostering of a better understanding of the duties and obligations of military doctors by combat personnel, military authorities and medical command, but also the promotion of the LOAC, in which enemy casualties are protected by their status as non-combat personnel. No distinctions between casualties are allowed, other than on medical grounds, and medical personnel “should not be forced to refrain from taking action”. Legal advisors specializing in the law of armed conflict are deployed in Barkhane to advise the military command on questions of international law, and to remind personnel of their duties before operations, which is a first step toward promoting LOAC awareness. This measure seems insufficient however, and a larger program of awareness training in medical ethics on overseas operations seems necessary. Current operational training before overseas deployment involves combat first aid training or simple reminders of the LOAC. While the LOAC represents an ethical basis for military doctors and a framework to avoid deviant behaviours, ethical reflection fits into a larger context involving many other factors. Knowledge and awareness of medical dilemmas, and training in ethical reflection using different methods described in the literature such as the principlist and the four quadrant approaches, may help doctors overcome these dilemmas [28, 29]. The benefit would be not only to facilitate ethical discussion and establish treatment conditions consistent with doctors’ values, ethical and LOAC-based, but also to limit the moral stress induced by ignored or unresolved dilemmas.

Several armies have already integrated ethical discussions on the possible dilemmas faced by healthcare professionals in the field into their training programs for overseas operations [48–50]. Operational preparations for French medical teams could be used as an opportunity for applied ethics in the form of moral case deliberation, to promote the ethics of discussion between healthcare personnel on the one hand and between healthcare and combat personnel on the other hand [51].

The different clinical vignettes described by participants in this study could serve as pedagogical material for operational preparations for ethics in overseas missions. In practice, this could involve discussing the dilemmas as part of medical simulation sessions to promote the ethics of discussion. More generally, the aim is to foster the ethical culture of the SSA for all personnel by creating an easily accessible online platform providing a list of anonymized and annotated case reports, with additional pedagogical material, as developed by the Swiss Center for Military Medical Ethics or by the British army [52, 53]. This material would be open not only to all members of the SSA, but also to combat personnel and the respective chains of command.

Several other measures could be considered to improve training in ethical thinking and develop the ethical culture of the SSA. The first would be to create a medical-military ethics think tank in charge of coordinating teaching, promoting research and keeping SSA personnel up to date with the literature. This group would be made up of SSA doctors and paramedical staff and researchers and academics working on medical ethics. Another objective could be to encourage joint ethical reflections between NATO members. The NATO Centre of Excellence for Military Medicine, an official “organisation responsible for assisting the Alliance in its goal of continuous transformation in the medical field”, could help coordinate the various armies’ activities on this topic by offering meetings and courses on medical-military ethics [54].

## Limitations

This was not an investigation to determine specific events during the deployment of the SSA in overseas operations, but a qualitative study based on subjective data from participants’ experiences. It provides an overview of what participants felt about their own experiences. The nature and the aims of the study were stated in the information provided to participants so there may be a certain level of voluntary bias in their responses. Self-selection bias cannot be excluded. We are nevertheless confident that saturation was achieved. The semi-structured nature of the interview guide may have had a framing effect that may have influenced how participants responded, especially regarding their dual loyalty. Furthermore, the translations into English of participants’ verbatim quotations may not perfectly reflect the original French content.

## Conclusion

This study provides unprecedented insight into the ethical dilemmas specifically encountered by a sample of French military doctors recently deployed in the Sahel as part of Operation Barkhane. The situations reported only involved the treatment of PUC and civilians, not French military personnel or those of partner forces. The question of MAC was widely brought up, in terms of the choice of patients to treat in a context of limited resources, but also in terms of the use of care as a means to an end by the military establishment, at the expense of patients’ interests. Disparities in the quality of care provided to French personnel and other patients because of the lack of follow-up facilities for the latter was also reported as creating ethical dilemmas. Operational training for French military doctors for the inevitable ethical issues they will face needs to be developed. The clinical vignettes presented in this study will be used to set up a specific pedagogical program on ethical dilemmas for SSA and combat personnel due to be deployed overseas.

## Data Availability

The verbatim transcripts have been withheld for security reasons (they may contain information on serving military personnel). Requests for access should be addressed to the French Military Health Service.

## Abbreviations

DMED: Director of medical affairs
GP: General practitioner
LOAC: Law of armed conflict
MAC: Medical assistance to civilians
MINUSMA: *Mission multidimensionnelle intégrée des Nations unies pour la stabilisation au Mali* (United Nations Multidimensional Integrated Stabilization Mission in Mali)
NATO: North-Atlantic treaty organization
NGOs: Non-governmental organisations
PECC: Patient evacuation coordination cell
PUC: Persons under control
SP: Specialist doctor
SSA: *Service de santé des armées* (French Military Health Service)
